# Draft Genome Sequence of Isoproturon-Mineralizing *Sphingomonas* sp. SRS2, Isolated from an Agricultural Field in the United Kingdom

**DOI:** 10.1128/genomeA.00569-15

**Published:** 2015-05-28

**Authors:** Tue Kjærgaard Nielsen, Sebastian R. Sørensen, Lars Hestbjerg Hansen

**Affiliations:** a Department of Environmental Science, Aarhus University, Roskilde, Denmark; b Department of Geochemistry, Geological Survey of Denmark and Greenland (GEUS), Copenhagen, Denmark

## Abstract

*Sphingomonas* sp. SRS2 was the first described pure strain that is capable of mineralizing the phenylurea herbicide isoproturon and some of its related compounds. This strain has been studied thoroughly and shows potential for bioremediation purposes. We present the draft genome sequence of this bacterium, which will aid future studies.

## GENOME ANNOUNCEMENT

The phenylurea herbicides have been used extensively for over half a decade and are applied in both urban and farming areas. They are important contaminants of water resources and are potentially harmful to animals that come into contact with them (1). *Sphingomonas* sp. SRS2 was isolated from enrichment cultures, originating from a British agricultural soil with a history of isoproturon (IPU) exposure, and was shown to be capable of IPU mineralization (2). Furthermore, strain SRS2 has been shown to be able to work well in coculture, survive in soil, and colonize rhizosphere (3, 4). The genes encoding the initial step of phenylurea herbicide degradation has been shown to be part of a conserved putative transposable element that appears in several sphingomonads from around the world (5). An *in situ* study showed that proliferation of strains related to *Sphingomonas* sp. SRS2 was correlating with degradation of isoproturon in soil (6).

*Sphingomonas* sp. SRS2 was streaked on an R2A plate (Difco Laboratories, Detroit, MI) to check for purity and incubated at 20°C for 18 days. A single colony was picked for DNA extraction using an UltraClean microbial DNA isolation kit (MoBio Laboratories, Inc., Carlsbad, CA, USA). Whole-genome sequencing libraries were prepared with the Nextera XT DNA sample preparation kit and sequenced on the Illumina MiSeq platform with the MiSeq version 2 reagent kit (Illumina, San Diego, CA, USA). Sequencing yielded 457,350 read pairs which were subjected to quality trimming, sequence contamination removal and merging of overlapping reads with Cutadapt v1.6 (7) and AdapterRemoval v1.5.2 (8). Trimmed reads were assembled with SPAdes v3.5.0 (9) with an estimated coverage of 25×. The final assembly was evaluated with QUAST v2.3 (10) and contains 178 contigs, of which 159 are larger than 1,000 bp constituting a total size of 4.63 Mbp. The G+C content of the assembly is 63.9%, and the *N*_50_ is 59,796. Contigs smaller than 200 bp were removed, and the draft genome assembly was uploaded to GenBank and automatically annotated with the NCBI Prokaryotic Genome Annotation Pipeline (PGAP), which predicted 4,317 coding sequences along with 51 RNA-encoding genes. The 1,493-bp 16S rRNA gene was identified and found to be 98% similar to that of *Sphingomonas wittichii* RW1 (accession no. NR_074268.1), using BLASTn (11). As previously determined, *Sphingomonas* sp. SRS2 harbors the genes *pudmAB*, which are associated with the initial *N*-demethylation step of phenylurea herbicide degradation in sphingomonads (5). These genes were confirmed to be present in the draft genome.

### Nucleotide sequence accession numbers.

This whole-genome shotgun project has been deposited at DDBJ/EMBL/GenBank under the accession no. LARW00000000. The version described in this paper is version LARW01000000.
